# Dr. Albert Brown Robinson

**Published:** 1908-05

**Authors:** 


### OBITUARY

---

Dr. Albert Brown Robinson, of Boston, died at his home on Blue Hill Avenue, Roxbury, March 30, 1908, aged 73 years. He graduated at the University of Buffalo, medical department, in 1857, and served during the civil war, first as assistant surgeon of the 10th and then surgeon of the 42d regiments of Massachusetts volunteer infantry. He was at one time dispensary physician of Roxbury and professor of surgery in the New England Female Medical College. It was understood that Dr. Robinson was the oldest practising physician in Boston.

